# Compassion focused therapy for self-stigma and shame in autism: a single case pre-experimental study

**DOI:** 10.3389/fpsyt.2023.1281428

**Published:** 2024-01-08

**Authors:** Marie Riebel, Agata Krasny-Pacini, Rumen Manolov, Odile Rohmer, Luisa Weiner

**Affiliations:** ^1^Laboratoire de Psychologie des Cognitions (LPC, UR 4440), Université de Strasbourg, Strasbourg, France; ^2^Centre d’Excellence STRAS&ND, Strasbourg, France; ^3^Institut Universitaire de Réadaptation Clémenceau, Hôpitaux Universitaires de Strasbourg, Strasbourg, France; ^4^Inserm U1114, Hôpitaux Universitaires de Strasbourg, Strasbourg, France; ^5^Faculty of Psychology, University of Barcelona, Barcelona, Catalonia, Spain; ^6^Hôpitaux Universitaires de Strasbourg, Pôle de Psychiatrie, Santé Mentale et Addictologie, Strasbourg, France

**Keywords:** autism, self-stigma, self-compassion, CFT, shame, compassion focused therapy

## Abstract

**Introduction:**

Exposure to public stigma can lead to the internalization of autism-related stigma (i.e., self-stigma), associated with negative health, occupational and social outcomes. Importantly, self-stigma is linked to shame and social isolation. Although elevated self-stigma has been reported in autistic adults, to the best of our knowledge, interventions designed to target this issue are lacking. Compassion is an effective way to reduce the emotional correlates of self-stigma (i.e., shame) and their impacts on mental health. However, no study has investigated whether compassion focused therapy (CFT) can effectively reduce self-stigma in autistic adults. The present study aims at investigating whether and how self-compassion improvement following CFT may reduce self-stigma and shame in an autistic individual.

**Methods:**

A single case pre-experimental design (SCED) was used with weekly repeated measures during four phases: (i) pure baseline without any intervention (A), (ii) case conceptualization (A’), (iii) intervention (B) where CFT was delivered, (iv) follow-up without intervention (FU). The participant is a 46-year-old autistic man with high self-stigma and shame. Self-report measures of self-compassion and self-stigma and a daily idiographic measure of shame were used.

**Results:**

There was a large increase in self-compassion between pure baseline (A) and the intervention phase (A’B) (Tau-U = 0.99), maintained at follow-up. Similarly, there was a moderate decrease of self-stigma (Tau-U = 0.32). In contrast, when we compared the whole baseline phase AA’ (i.e., considering the conceptualisation phase as baseline) to the intervention (B), there was no change in self-stigma (Tau-U = −0.09). There was no change in self-stigma between the intervention (B) and follow-up (Tau-U = −0.19). There was a moderate decrease in daily shame reports between the baseline (AA’) and the intervention (B) (Tau-U = 0.31) and a moderate decrease between the pure baseline (A) and intervention phase (A’B) (Tau-U = 0.51).

**Conclusion:**

CFT was feasible for this autistic client and our results show that CFT led to the improvement of self-compassion. Changes on self-stigma measures were moderate. Self-stigma may need more time to change. Because self-stigma is involved in poorer social functioning and mental health in autistic adults, our results are promising and suggesting conducting more large-scale studies on CFT in autistic adults.

## 1 Introduction

Autism spectrum disorder (ASD) is defined as a neurodevelopmental condition, characterized by difficulties with social communication and interaction, altered sensory processing and patterns of repetitive behaviors and intense interests (1). Recent worldwide estimations of autism prevalence indicates that approximately 1% of the population is autistic (2). Importantly, lifetime prevalence of anxiety disorders and depressive disorders in autistic adults are particularly high, ranging from 23 to 42% (3). This elevated prevalence of mental health difficulties in autistic people is thought to be partially explained by the high levels of bullying they experience (4–6) and by the frequent experience of non-acceptance from others (7, 8). Such discriminative behaviors, probably linked to autism-related public stigma, can increase the likelihood of using camouflaging behaviors to pass as non-autistic (e.g., masking) (9), which is associated with poor mental health and increased isolation in in autistic people (10, 11).

The process of stigmatization encompasses three facets, contributing to the discrimination experienced by targeted groups. The first one, public stigma, refers to the negative attitudes, beliefs, and stereotypes held by the general population towards individuals or groups who are perceived as different or deviating from societal norms (12); affiliative stigma designates the prejudice and discrimination experienced by individuals who are closely associated with a stigmatized person or group (13); finally, self-stigma refers to the internalization of societal stereotypes and negative beliefs by individuals who belong to a stigmatized group (14). Importantly, self-stigma has been shown to lead to a decrease in self-esteem, self-efficacy, and a heightened feeling of shame or self-blame (14).

The relationship between self-stigma, shame and poor self-esteem may be explained by the social mentality theory (SMT; (15)). SMT conceptualizes stigma as a social threat that challenges the social ranking of the stigmatized individual, engendering feelings of inferiority (15). Thus, the perception of being on a lower social rank, akin to self-stigma, can provoke feelings of shame (16). Shame is defined as a self-conscious emotion, involving worthlessness, powerlessness, and isolation. Usually viewed as one of the most intense and incapacitating self-conscious emotions, shame can be understood as a socially focused emotion, cued by threats to the social self or one’s status (17). Given its socially threatening nature, shame comes with a willingness to escape the situation, hide, or conceal deficiencies (18–20). Thus, shame is seen as the emotional consequence of self-stigma, linking stereotypes to behavioral consequences (e.g., social isolation) (21).

While numerous studies have investigated self-stigma associated with mental illness, studies concerning self-stigma in autism are scarce (22–26). This may seem surprising given the high prevalence of self-stigma in autistic adults, which ranges between 15% (22) and 45.2% (27) according to recent estimations. Furthermore, few studies have investigated shame and its relationship with self-stigma in autistic people. In a recent study led by our team in a sample of 689 autistic adults, self-stigma was found to be highly correlated to shame and shame mediated the relationship between self-stigma and depression (27). These results highlight the need to develop and evaluate specific interventions to target shame and self-stigma in autistic adults in order to mitigate the negative outcomes associated with the internalization of autism-related public stigma (e.g., social isolation and depression). Yet, to the best of our knowledge, interventions targeting shame and self-stigma in autistic adults are lacking.

Self-compassion has been recently put forward as a potential buffer of the effects of public stigma on self-stigma and mental health (28). Self-compassion is defined as kindness and support towards oneself when experiencing suffering (29). Self-compassion involves responding to life’s difficulties in three specific ways: (i) kindness as opposed to self-judgement, (ii) mindfulness as opposed to over-identification to painful emotions and thoughts, and (iii) common humanity as opposed to isolation, that is, perceiving one’s suffering as an integral part of the human experience. In non-autistic samples, self-compassion has been strongly associated with numerous health benefits such as higher levels of happiness and well-being, better sleep quality and lower levels of depression, anxiety, stress and self-harm (30–33). In the context of self-stigma, increasing self-compassion may therefore contribute to the reduction of self-blame and act as a buffer of the negative effects of public stigma, by facilitating social resources and increasing the willingness to ask for help (28). In addition, self-compassion may foster self-perspectives that are more balanced, first, accepting both positive and negative aspects of self and, second, learning how to observe and let go of self-stigmatizing thoughts, emotions and behaviors. This may, in turn, mitigate the negative outcomes associated with self-stigma (34).

Some empirical results provide support to Wong et al.’s (28) model, which highlights the protective role of self-compassion. For instance, a correlational study found that self-compassion partially mediated the relationship between self-stigma relative to one’s weight and negative health outcomes (e.g., somatic symptoms and quality of life) (34). Similarly, in another study, self-compassion was found to moderate the impact of HIV-stigma and negative affect (35). Furthermore, self-compassion has been found to moderate the relationship between public stigma and the anticipated self-stigma of help seeking when one has a mental health problem (36).

Despite the elevated prevalence of self-stigma and mental health difficulties in autistic individuals, few studies have investigated self-compassion in relation to autism. Recently, two online studies found that autistic participants reported significantly lower self-compassion levels than non-autistic adults. Interestingly, in both groups, those with higher levels of self-compassion had fewer depression symptoms (37, 38). Moreover, autistic traits and self-compassion in both autistic and non-autistic individuals have been found to be negatively correlated (38), suggesting that low levels of self-compassion are related to the cognitive style found in autism (e.g., social difficulties and cognitive inflexibility). Relatedly, Cai and Brown’s (39) review paper suggested that self-compassion may improve mental health in autistic adults through the modification of emotions (as an emotion regulation strategy). Consistent with this view, empirical results by Cai et al. (40) demonstrated that emotion regulation mediated the relationship between self-compassion and anxiety/depression in autistic adults. Thus, it is likely that self-compassion may help autistic adults regulate their feeling of shame associated with self-stigma.

Interestingly, self-compassion has been recently found to moderate the relationship between self-stigma and depression in autistic adults (27) suggesting that an intervention aiming at increasing self-compassion might be useful for reducing self-stigma. In particular, compassion focused therapy (CFT) seems relevant in the context of self-stigma as it has shown its efficacy for targeting shame and hostile self-to-self relationship (41). CFT is a biopsychosocial, evolution-informed psychotherapeutic approach that builds on traditional cognitive behavioral therapy (CBT) principles and blends empirical knowledge from affective neuroscience, social and developmental psychology (16). Theoretical benchmarks in CFT includes an understanding of how evolutionary processes have shaped our minds and brains to serve a variety of functions. CFT focuses particularly on *social mentalities,* defined by Gilbert (42) as patterns of brain activity organizing our relationships and social roles by shaping different parts of our minds, i.e., motives, emotions, cognitions and behaviors. Relevant examples of social mentalities are the caregiving and care-receiving mentalities in contrast to threat-giving and threat-receiving social interactions. This understanding of social mentalities is relevant to the experience of stigma which can be understood as a social rank (dominant-subordinate) relationship. Because CFT provides an understanding of the function of threat-based processes, specifically when threat-giving and threat-receiving social rank mentalities have been internalized and used to interact with oneself, this therapeutic approach seems particularly relevant to target self-stigma. Moreover, CFT has gathered a large body of evidence in the treatment of shame and self-blame in a wide range of clinical settings (43, 44). Given its focus on the psychological processes and affective aspects that can be found in self-stigma, CFT may be more effective than existing interventions for the reduction of self-stigma and shame (45). CFT has not yet been evaluated in autistic individuals. However, a recent paper has provided strong theoretical support for the clinical relevance of CFT for the treatment of shame-related problems experienced by autistic adults (46). Hence, through its focus on self-compassion, CFT is likely to improve mental health by specifically targeting shame and self-blame (27).

The present study aims at investigating whether and how self-compassion improvement following CFT may reduce self-stigma and shame in an autistic individual. To do so, using a single case pre-experimental design, we will investigate whether and how scores of self-compassion, self-stigma and shame measures evolve following CFT in an autistic adult presenting with high levels of self-stigma. Specifically, we hypothesize that self-compassion scores will increase post-therapy, while self-stigma and shame scores will decrease. Single-case experimental designs constitute methodologies of growing interest in rehabilitation settings. These methodologies are recognized as relevant to investigate parameters related to the efficacy of a new intervention in a small number of participants before running a larger group trial (47).

## 2 Materials and methods

### 2.1 Design

To be included, the participant had to be autistic, present with an elevated score on the internalized stigma of mental illness (ISMI-9) (>2.5; (48)), have an IQ within the normal range, and be willing to participate in CFT. The participant was recruited from the University Hospital of Psychiatry clinics following his participation in a dialectical behavior therapy (DBT; (49)) program. A single case pre-experimental design (SCED; (50)) was used with weekly repeated measures of self-compassion and self-stigma as well as a daily ideographic measure of shame, during four phases, i.e., (i) pure baseline, (ii) conceptualisation, (iii) active compassion focused therapy, (iv) follow-up. Table 1 provides a detailed description of the contents and the duration of each phase. Using the baseline as a benchmark, the participant functions as his own control and the primary analysis is a comparison of weekly measures during the baseline, and the subsequent phases. The conceptualisation phase consisted of individual sessions with the participant to establish a working therapeutic alliance, identify goals and conceptualize his difficulties according to the CFT formulation. The active intervention phase consisted of 20 weekly individual CFT sessions. Throughout the study duration, the participant responded to weekly measures of self-compassion and self-stigma and daily measures of shame through an online journal that he chose to design (instead of using a paper survey). All sessions were conducted by a clinical psychologist trained in CFT. Ethical approval was assigned by the French ethics committee (Comité de Protection des Personnes Nord Ouest II) - (2022-A02501-42). Written informed consent was obtained from the participant for the publication of any potentially identifiable images or data included in this article.

**Table 1:** Description of phases.

	Baseline		Intervention phase	Follow-up
	A: Pure baseline	A’: Case conceptualization	B: Active CFT	FU: Follow-up
Contents	No sessions with therapist.	Sessions with therapist consisted of assessment, formulation, therapeutic relationship, safety and safeness, tasks and goals.	Sessions with therapist consisted of CFT for self-stigma including elements of compassion mind training and working with shame and self-stigma using the compassionate self.	No sessions with therapist.
Number of weeks	5	4	20	5

### 2.2 Measures

Acceptability of the intervention was measured via the assiduity in sessions and his adherence to in-between sessions practices. The participant designed an online journal which was shared with the therapist where he took notes of his home practices.

#### 2.2.1 Self-Compassion Short Scale

Self-compassion was measured with the Self-Compassion Scale (51) in its short version (52). The French validation of the scale in its long version indicates good psychometric properties (Cronbach α = 0.94) (53) consistent with the results of the English short version of the scale (Cronbach α = 0.87) (52). The scale consists of 12 items. The responders are asked to indicate how often they act toward themselves in difficult times using a Likert-scale ranging from 1 (“almost never”) to 5 (“almost always”). For example, item 2 states “I try to be understanding and patient towards those aspects of my personality I do not like.” Only total scores were used in this study as recommended by Raes et al. (52). The total score was calculated as a total mean after having reversed coded the negative subscale items (self-judgment, isolation, and over-identification).

#### 2.2.2 Internalized Stigma of Mental Illness (ISMI-9)

Self-stigma was measured using the Internalized Stigma of Mental Illness Scale (ISMI) in its short 9-item version (54), it is an abbreviated version of the full 29-items designed to assess self-stigma among persons with psychiatric disorders (48). The scale is a self-report instrument with each item rated on a 1 (strongly disagree) to 4 (strongly agree) Likert scale. According to Ritsher and Phelan (48) a mean total score of >2.5 indicates high levels of self-stigma.

#### 2.2.3 Daily reports of the feeling of shame

To further inspect the variations of feelings of shame on a daily basis, participant and researcher collaboratively decided to design a question aiming to assess shame. It was collaboratively decided to use the following question: *How often have I felt ashamed today?* The participant rated his feeling of shame on a Likert scale ranging from 1 (never) to 5 (most of the day).

### 2.3 Statistical analysis

The total scores calculated from the weekly measures of self-compassion and self-stigma were graphically displayed. Visual analyses focused on the following data features: level, trend, variability and overlap of data points (55). No visual aids were used in the process of visual inspection of the graphed data. The visual analysis of weekly measures were complemented with the use of statistical indicators. Tau was used when it was visually evident that there is no improving baseline trend, whereas Tau-U with baseline trend control was used when the visual analysis suggested the need to control for spontaneous improvement during the baseline.

Tau-U (56) has several versions, the simplest of which also quantifies overlap, but in a slightly different way as compared to NAP: if NAP is expressed in a scale from 0 to 100 and Tau is expressed in a scale from 0 to 1, Tau = 2 * (NAP/100) − 1 (57). This quantification is appropriate when there is no improving baseline trend. Given that there was no such trend for the shame data (unlike the measurements of self-compassion and self-stigma), we used Tau. Another version of Tau, Tau-U, allows quantifying monotonic baseline trend and correcting for this trend when representing the amount of nonoverlap. Therefore, we used Tau-U to evaluate the changes in self-compassion and self-stigma.

In addition to Tau calculations, we also used the percentage of goal obtained (PoGO) for self-compassion and shame in order to quantify to what extend goals were achieved (58). The set goals in the context of the calculation of PoGO was set *a posteriori*. Regarding daily reports of shame, the goal was set to 1 indicating “*I did not feel ashamed today*” and regarding self-compassion, the goal was set to 2.5 which corresponds to the benchmark from low self-compassion to moderate self-compassion (51).

The following comparisons were made: (i) A versus A’B phase, i.e., assuming that the conceptualisation phase A’ is already an intervention because of the awareness and psycho-education it includes; (ii) AA’ versus B, i.e., considering that the conceptualisation phase is part of the baseline because no active CFT ingredients are delivered; (iii) A’B versus FU and (iv) B versus FU.

### 2.4 Case illustration

#### 2.4.1 Presenting problem and client description

Julian (a pseudonym) is a 46-year-old autistic man working as a free-lance web designer. He received an ASD diagnosis at age 41. In addition to ASD, Julian has been diagnosed with social anxiety and asthma. He currently takes no medication. Two years prior to his participation in CFT, he benefitted from DBT (59) which led to a significant reduction in self-harming behaviors and emotion dysregulation. However, Julian still struggles with a very depreciating self-image, feelings of inferiority and self-stigma since he has received the diagnosis of ASD (ISMI score of 3.3 at inclusion). Julian is divorced and has two children who are now adults. He lives alone and is involved in a romantic relationship. He reports suffering from loneliness and lack of friendships.

#### 2.4.2 Intervention

##### 2.4.2.1 Overview

To increase compassion for self, others, and the ability to receive compassion from others, the CFT therapist guides patients to develop feelings of warmth, safeness and soothing through compassionate mind training (16).

The intervention consisted of weekly, individual sessions of approximately 1 h. The content of the intervention was adapted from a group CFT program for self-stigma developed by our team, presented in Table 2 (45). However, since the therapy was conducted in an individual setting, it was possible to adapt the contents to individual situations. The twelve modules of the program were conducted in 20 individual sessions. This program was built based on core CFT psychoeducation components and core experiential practices such as compassion focused imagery, chair work and letter writing. Details of the therapeutic sessions can be found in Table 2. Each session started with a soothing rhythm breathing practice and contained psychoeducation elements, socratic dialogue around a theme and in-session experiential practices such as compassionate imagery, role plays and chair work. The overall aim of the CFT program is to help the patient shift from a hostile and critical self-to-self relationship to a more compassionate relationship to self. Indeed, the participant develops a compassionate identity through which he can respond to parts of oneself that might suffer. During the therapy process, the clinician adapted CFT exercises to fit the interests of the participant (60). For example, the participant’s interest in writing was used to create dialogues between different selves based on the CFT framework.

**Table 2:** Content of modules and home practices of the COMPASS program (45).

Modules	Session title	Session content	Home practice
1	Welcoming and creating a safe place Definition of compassion and personal goals	Reflection on a safe place agreement for the therapy Exploration of what is (and what is not) compassion Short introducing to Soothing Rhythm Breathing (SRB)	Soothing rhythm breathing (SRB) (https://youtu.be/Md2c0h6bogE)
2	Compassion wisdom: the tricky brain and the social construction of self	SRB Tricky brain problem How and why we are different to other animals: our unique capacity for self-consciousness and self judgement (“not our fault”) We are only one version of the infinite possible versions of self Understanding the influence of our social environment on our construction (“not our fault”)	Soothing rhythm breathing (SRB) Identifying my own tricky brain loops
3	Compassion wisdom: Three emotional regulation systems	SRB Introducing the three circles model: threat, drive and soothing Evolutionary function of emotions	Soothing rhythm breathing (SRB) Drawing my three circles and identifying triggers
4	Compassion wisdom: stigma and self-stigma	SRB Introduction stigma and self-stigma Understanding the path from public stigma to self-stigma (“not our fault”) through the social construction of self and the tricky brain Consequences of self-stigma through the lens of the 3-circle model	Soothing rhythm breathing (SRB) Filling the self-stigma model and tricky brain loops associated
5	Compassionate engagement: thinking, imagery and body postures can influence our physiology	SRB Introducing the mindfulness circle Thoughts and imagination can impact our physiology: experiencing with attention, postures, tones of voice, SRB Safe place imagery Ideal compassionate other imagery	Safe place imagery (https://youtu.be/Md2c0h6bogE)
6	Compassionate engagement: the compassionate self	Experiencing with the compassionate self (postures, tone of voice, feelings of warmth, actions)	Compassionate self-imagery (https://youtu.be/1KELVnBvvho)
7	Compassionate courage: multiple selves	Embodying the compassionate self to respond to the threat system thoughts and emotions	Compassionate self-imagery (https://youtu.be/1KELVnBvvho)
8	Compassionate courage: how to respond to the self-stigmatizing self	Exploration of self-stigma and self-critic: reasons to be and consequences Using compassionate self to respond to self-stigma	Compassionate self-imagery (https://youtu.be/1KELVnBvvho)
9	Compassionate courage: dealing with difficult emotions	Understanding of shame and guilt Responding to difficult emotions with compassion	Embodying compassionate self in everyday life
10	Compassionate courage: compassionate assertiveness	Understanding the components of compassionate assertiveness compared to submissive and aggressive expression Practicing compassionate assertiveness through role plays	Compassionately asking something we need
11	Compassionate courage: cultivating the compassionate self	Writing a compassionate letter Sharing of compassionate letters	Compassionate letter
12	Continuing my journey with compassion	Building my personal compassionate tool bag Plans for continuing practicing compassion Gratefulness and compassion wish	

##### 2.4.2.2 Case formulation (phase A’)

The CFT case formulation is evolution-informed and helps creating a de-shaming and de-pathologizing understanding of one’s difficulties. Specifically, psychoeducation in CFT involves elucidating the evolutionary origins of our brains, helping clients recognize that our brains are tricky and reassuring them that they are not to blame (61). Brain functions that were advantageous for our survival over millions of years can now pose considerable difficulties in our daily lives. For instance, our brains possess an inherent tendency to be hyper-aware of threats, guided by the principle “better safe than sorry.” For a more comprehensive understanding of the evolution-informed psychoeducation in CFT, see Gilbert (62) for a review. Case conceptualization in CFT comprises an evolution-informed psychoeducation and entails an understanding of one’s difficulties within this functional framework.

**Figure 1:**
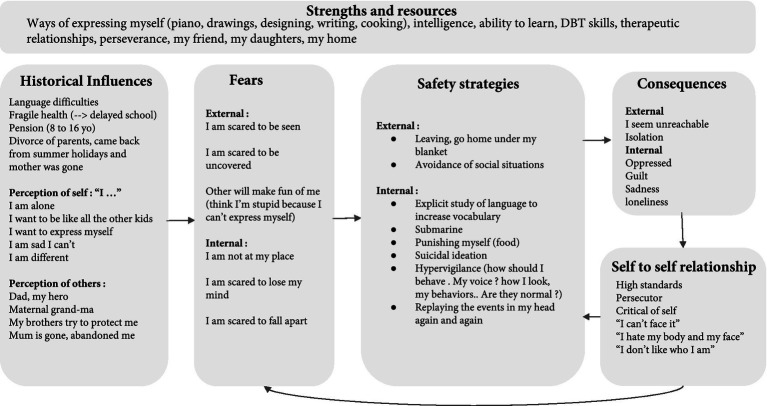
Conceptualization of Julian’s difficulties.

Using Socratic dialogue, the *fundamental fears* of Julian, consisting of external fears (i.e., in relation to how others perceive oneself; e.g., “fear *that others will make fun of me and reject me*”) and internal fears (i.e., fears related to one’s own perception of oneself; e.g., “*I am scared to fall apart*”) were normalized through the exploration of *historical influences* (see Figure 1). In particular, the therapist explored early memories with an emphasis on experiences of warmth and safeness as opposed to memories of feeling threatened and ashamed. For example, as a child, Julian had a speech impediment which led to school bullying; he felt excluded and ashamed. Julian also reported the loss of his feeling of safeness following the divorce of his parents, as his mother left home while he had been sent away for holidays during the summer. Concerning feelings of warmth, Julian felt safety around his father and grandmother. Julian developed feelings of inadequacy and loneliness that might be explained by the school bullying he experienced and by the abrupt departure of his mother. Safety strategies were then identified as behaviors used to avoid or escape situations that may bring about his fundamental fears and other painful emotions. For instance, Julian often escaped social situations and fled home to find a place where he felt safe. Internal protective reactions include hypervigilance regarding his social behaviors (e.g., ruminations over how to react to prevent rejection and mockery from others). During the conceptualization phase, the therapist normalized these safety strategies as they were viewed through the lens of their protective functions. For example, monitoring his behaviors might have prevented further harm in the past (e.g., from bullies at school). However, these safety strategies have led to unintended consequences, e.g., fatigue. Interestingly, fatigue may be related to camouflaging, which is defined by Hull et al. (63) as coping strategies including “explicit techniques to appear socially competent and finding ways to prevent others from seeing their social difficulties.” Camouflaging is highly prevalent in autistic adults and is associated with fatigue and negative mental health outcomes (64, 65). All of these can fuel the development of high standards towards oneself and a self-critical self-to-self relation. Indeed, Julian felt “*bullied at school and now I am my own persecutor*.” One of the purposes of collaborative case conceptualization is to de-shame those unintended consequences. Consistently, the CFT framework employs the terminology “*safety behaviors*” instead of “*dysfunctional behaviors*” to acknowledge that the patient is doing their best to feel safe. In other words, the patient is doing their best given the social construction of the self and the way the human brain evolved from an evolutionary perspective (i.e., in CFT, the human brain is called the “tricky” brain). In addition to the specific components of the CFT case conceptualization, the strengths of the participant were highlighted (66). The latter factors were related to important values such as creativity and love for the arts.

Following the case conceptualization, three therapeutic goals were identified: i.e., “be less self-critical towards and accept my imperfections,” “start writing my novel,” “connect with people.”

##### 2.4.2.3 Compassion focused therapy (during phase B)

The 20 therapy sessions were based on the group program developed by our team (Table 2). The program is a step-by-step manualized CFT treatment targeting self-stigma. In this study, the same treatment contents were applied, albeit more flexibly, to adapt to the participant’s individual needs. The primary objective of the intervention was to assist the client in cultivating a caregiving and care-receiving mentality, which involved nurturing a compassionate mind or compassionate self. The compassionate self served as a foundation for the client to effectively respond to his emotions and experiences rooted in threat. Thus, the patient sought to foster his compassionate self, equipping himself with the necessary qualities and skills to incorporate compassion into his relationship with himself and with his recently discovered autistic identity. The intervention is illustrated through the presentation of some key therapeutic practices below. Based on the key adaptations suggested by Keenan et al.’s (60) first-hand account of DBT, the main adaptations of CFT for this specific person consisted of connecting the therapeutic tasks to his specific interests, that is, the arts (literature, classical music, and visual arts). Hence, the key practices outlined below were developed based on Julian’s interests. Also, we provided Julian with a therapy booklet containing visual summaries of key psychoeducational contents and worksheets to guide him with his home practices.

###### 2.4.2.3.1 First session: getting acquainted with compassion

Julian initially associated the words “*altruism, non-judgment, openness, empathy*” with compassion. After validating this intuitive wisdom, we explored the meaning of compassion through the recall of a memory where he felt willing to help someone. We specified the two psychologies of compassion, i.e., (i) sensitivity to suffering in self and others and (ii) engagement to alleviate and prevent suffering (16). Julian then provided a recent example where he acknowledged that he could understand the person’s difficulties from a cognitive standpoint, which he called “*external knowledge*,” but he explained how difficult it was to have “*internal knowledge*,” that is, feel compassion. A metaphor to explain the difference between knowing and feeling was then used based on Julian’s practice of music: “*I am a pianist, I could play cello or doublebass, I know how music works, but I am not a cello or doublebass player, I cannot feel the music on cello like I feel it when I play piano*.” This highlighted that one can practice feeling compassion just like one can learn to play a new instrument. At first, it might be difficult and require a deliberate effort but, with training, compassionate feelings may emerge. This first session helped to reinforce Julian’s engagement in the therapy and his willingness to cultivate compassion.

###### 2.4.2.3.2 Cultivating the compassionate self

Compassionate mind training is an essential component of CFT (67). To cultivate the evolved social mentality for caring and compassion, different practices with breathing, body posture, voice tone, and imagery were used. The objective was to build and train a compassionate self, able to give compassion to one’s own suffering.

The first CFT technique used was Soothing Rhythm Breathing (SRB). SRB allows to experiment the connection between bodily sensations, emotions, and mental processes; this in turn helps to regulate arousal (see (68) for more details about SRB). SRB is a breathing method that permits to decrease respiration frequency and increase heart rate variability (HRV) (69). To do so, it was important that SRB was experienced as a technique that goes beyond mere relaxation; instead, it is aims to function as a cue to activate the soothing system while simultaneously regulating the threat system (70). Indeed, greater HRV is linked with reduced negativity bias and an enhanced willingness to embrace novelty (71). In Julian’s case, a cognitive shift through SBR was reported after his second independent practice with this breathing method “*in a time of stress when many thoughts were present*,” “*through breathing, the thoughts left, slowing down, as if we were stopping time*.” After the first introduction of SRB, each session started with a SRB practice, which was progressively complemented with new learnings such as a soft internal voice tone or a friendly facial expression. Indeed, SRB constitutes the initial phase of most therapeutic tasks in CFT (68, 72). We used posture and breathing exercises to establish physiological safeness, fostering a compassionate mind. In session 3, Julian reported that he had turned to SRB spontaneously during a difficult situation where he felt tense and angry while he was driving home. Instead of stopping on the side of the road and taking a nap to calm down, he “…*used the vibrations on the steering wheel as an anchor that helped me to calm down*.” He highlighted that this was very effective and soothing: “*this strategy of calm through breathing, while driving, is less risky than stopping on the side of the road to sleep*.” Thus, there was generalization of new learning and SRB became a helpful strategy to regulate intense emotions. Based on the intuitive wisdom Julian showed in this situation, the therapist suggested that he could use the same strategy during other difficult situations. For instance, in the city, if intense tension arises, Julian usually sits down and plugs his ears. He dislikes this behavior and chose to practice SRB instead while holding a small object of his choosing (i.e., a specific rock), to feel “anchored’ akin to the feeling he reported while touching the steering wheel. As the weeks went by and Julian continued to regularly practice SRB, he shared in week 21 (10th session of active CFT) “*I know it’s there, I can activate it when I need*” and “*there is a part of me in this rock, the part that is wise and compassionate*.”

Another fundamental technique in CFT consists of training the caring system through compassion focused imagery (e.g., compassionate memories, visualizing caring individuals, creating a safe place, imagining a compassionate color and a compassionate ideal person). These practices were first conducted in session and debriefed with the therapist who targeted the sensations and feelings during the practices. Julian was able to experiment further at home through audio recordings of the sessions or with videos made by the therapist (73). These practices increased his familiarity with the feelings and sensations associated with compassion. To facilitate the implementation of these practices, we relied on Julian’s love for the arts. For example, Julian chose specific music pieces to accompany certain compassionate practices. His safe place was first chosen to be his grandmother’s house, which was associated with warmth and feeling of safeness but also a sense of loss, grief, and deep sadness. After different explorations of potential safe places, Julian thought of an arts foundation he used to visit with his grandmother; this safe place helped him feel warmth, a sense of safeness and openness and he felt welcomed there exactly for the person he is. Julian discovered that looking at a picture of the arts foundation helped him to feel peaceful. Between-sessions practices consisted of SRB and compassionate imagery. Examples of his personal notes regarding these between-session practices are presented in Table 3.

**Table 3:** Compassionate object interview.

Aims	Relating to aspects of self as if he was the compassionate object. This experience of self as an external person permits to transfer interpersonal behaviors and competencies to self. It can increase the client ability to mentalise and generate self-compassion by focusing on self from an external perspective (74).
Therapist questions	How long have you been in Julian’s life? What role do you have for Julian? What do you do for Julian? When does Julian turn towards you? When does Julian need you? Why are you so important for Julian? How do you feel about him? What do you wish for Julian? What would like him to know?

In CFT, training of the compassionate mind can include role plays, chair work and embodiment practices. These were particularly difficult for Julian at first as they tapped on one of his fundamental fears, i.e., “*others will make fun of me*.” To facilitate these practices, the therapist first asked Julian to choose an object that had special meaning for him and to bring it to the next therapy session. The therapist then interviewed Julian as he embodied the compassionate object (75). This allowed him to experiment embodiment practices in a safe and playful way as he embodied a toy from his childhood. This therapeutic task was also a way to experiment giving himself compassion through a third person perspective.

###### 2.4.2.3.3 The multiple selves

Since the beginning of the intervention, Julian was introduced to the concept of having multiple selves, where each emotion was treated as a separate entity. For instance, when he expressed feeling sadness, the therapist acknowledged this as the expression of his “sad self.” This approach aimed to foster a more relational perspective towards emotions, allowing the client to engage with them as distinct entities rather than becoming overwhelmed by them. Throughout sessions, the therapist guided Julian to identify, label and picture his different parts. The different selves were explored by the therapist with an attitude of curiosity. Here again, Julian was able to fully embody his different parts, e.g., “sad self,” “angry self,” and the therapist asked questions like “*Angry self, what would you like to say?*” “*how does the anger manifests in your body?*” “*What would you do if you could take control over Julian?*” This allowed Julian to become aware of different emotional parts within himself and to better understand the underlying motives and needs of each part. For example, we explored the “blaming self” that emerged during a difficult situation (i.e., Julian was not invited for the birthday of his friend) (*cf.* Table 4).

**Table 4:** Example of the blaming self.

The blaming self/“the spear”	
Sensations	Jaws are tensed, like having a spear through the chest
Emotion	Shame
Thoughts	I am not normal, and I deserve to be punished. I must isolate. I must harm myself
Behaviors	I cannot speak, urge to punch my hands into a wall
Underlying need	To be included and accepted

In CFT, a relational approach is used to explore the different parts of the self, akin to defusion described in mindfulness-based approaches (e.g., Acceptance and Commitment Therapy; (76)). In addition to defusion, in CFT, the therapist explores how the client would like to respond to the parts within himself to foster understanding and compassion. This allowed Julian to identify certain qualities (“*kindness, generosity, altruism, gentleness, understanding, tolerance and strength*”), which were then useful to guide him in constructing his compassionate self. This ideal compassionate self was further reinforced through imagery practices, role-playing and embodiment exercises, allowing him to immerse in the role and experience the compassionate attributes firsthand. Painful emotions were explored through the lens of the multiple selves and then integrated trough the compassionate self that validated the needs of each part.

###### 2.4.2.3.4 Bringing compassion into daily life

As Julian’s compassionate identity became more clear, other therapeutic tasks were included to embody his compassionate self in his daily life. This started at home while doing activities such as listening to music, playing the piano, or doing house chores. Julian deliberately changed his body postures and internal voice tone, mindfully observing his sensations, and looking at the world through the eyes of his compassionate self. After practicing at home, Julian was encouraged to practice outside. This was a stressful situation for Julian as he felt inadequate and feared the mockery and rejection of others. To embody his compassionate self, Julian shifted from the ashamed self to the compassionate self when going to the bakery and talking to the salesperson. He did so via the practice of SRB, intentionally changing his body posture and voice tone, he observed “*when I change my posture, others change too*.” He also noticed that he felt more comfortable and talked more during the conversation. He still felt anxious but instead of having the “blaming self / the spear” run the show and make him feel inferior, the compassionate self encouraged him. Julian acknowledged that “*Compassion is part of me now*,” highlighting how his compassionate self had been incorporated in his sense of identity.

###### 2.4.2.3.5 Compassion for the stigmatized self

Julian’s interest in writing was an important asset when the compassionate self was to give compassion to the stigmatized self. Indeed, one of the main practices of CFT is the compassionate letter writing (68). To do so, one writes a letter to oneself (i.e., the part who is suffering) through the lens of the part of the self who is wise, caring, and strong, i.e., the compassionate self. Before writing the letter, Julian first practiced SRB and immersed himself in a compassionate self-imagery. Then he identified the part of the self who was suffering, i.e., the stigmatized self, validated its suffering, shared his compassionate wisdom about humankind’s tricky brains (i.e., “even though it is not my fault, it is my responsibility”), and showed support and willingness to change. This practice cued an important fear of compassion, which led Julian to postpone its writing. Some time was taken during sessions to identify key fears and resistances and to compassionately respond to them. Motivational interviewing techniques were used to help him activate his compassionate self and start writing (77). During this process, Julian noted “*Even if I do not act, I can see and identify how I can make progress and I stop bullying myself*.” With the compassionate self, Julian wrote himself a letter that progressively became the novel he wanted to write for a long time (i.e., one of the therapy’s goal). In week 25, he felt that “*The therapy allowed me to move forward and feel safe. My writing project takes up more and more space*.” This highlights the importance in CFT of creating safeness and of cultivating the courage, through compassion, to achieve the goals of one’s life worth living.

## 3 Results

### 3.1 Acceptability

The participant did not drop out of therapy, nor did he miss any session. Julian completed an online journal shared with the therapist where he took note of his practices of soothing rhythm and compassion focused imagery. In total, during the compassionate mind training phase and follow-up, he wrote 125 entries about soothing rhythm practices and several compassionate imagery practices such as the “safe place” and “compassionate color.” In the journal entries, Julian wrote how he felt before, during and after the practices as well as any practice-related discoveries or comments. Example of journal entries of practices are presented in Table 5.

**Table 5:** Examples of Julian’s journal entries of compassion practices at home.

Week number and period	Type of practice	How I felt before	How I felt during	How I felt after	Comments
Week 13 Compassionate mind training	Soothing rhythm breathing	Very angry	The first minutes are difficult	Relieved, soothed, calm	
Week 14 Compassionate mind training	Safe place	Tired, angry	It is difficult to keep focus	A little bit calmer	I cannot seem to choose my safe place, several images come to my mind
Week 22 Compassionate mind training	Safe place	Tensed	The successive heat waves we are experiencing at the moment allow me to give more reality to this place. I’ve often been there when it was hot, and very quickly, flagrances of pine and stones splashed by watering invade my thoughts	Happy and at peace	Now that I’ve identified my place of serenity, everything is much simpler
Week 27 Compassionate mind training	Compassionate self	Tired	I feel good and happy to practice this	Peaceful	I’ve just come from a nature walk, I’m sitting on a bench in the sun
Week 44 Follow up	Soothing rhythm breathing	Wilful	I think back to Marie’s words before I left, and her encouragement, which for me was an expression of a certain kindness	I’m confident I’ll have a beautiful day	I came across Agnes Obel’s name in one of my music playlists, and I must confess to having been very moved by the memory of my first listen. This “incident” undoubtedly reconciled me with the practice of soothing breathing rhythms, the very thing I had long abandoned, so unable was I to find the answers to my inner suffering. Now I know that this name and the music behind it are like all the lights in the sky when the night is at its darkest

### 3.2 Self-compassion, self-stigma and shame

**Figure 2:**
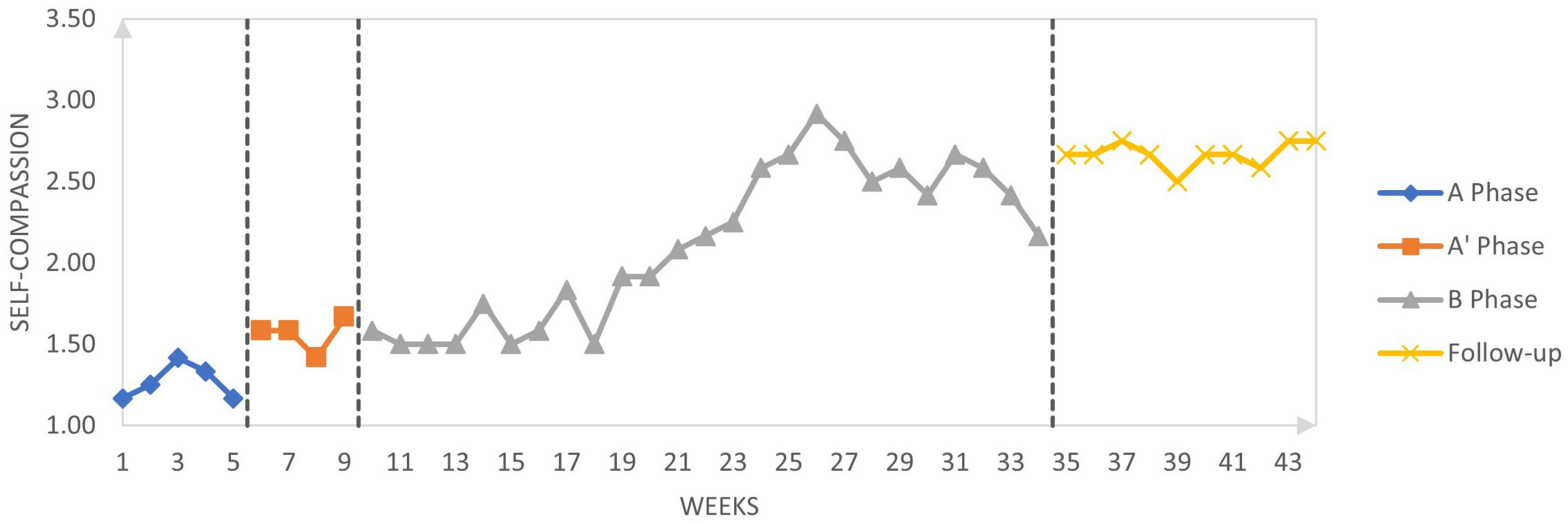
Weekly self-compassion scores across phases.

**Figure 3:**
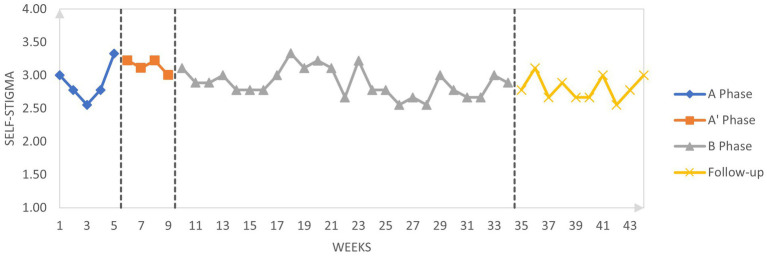
Weekly self-stigma scores across phases.

**Figure 4:**
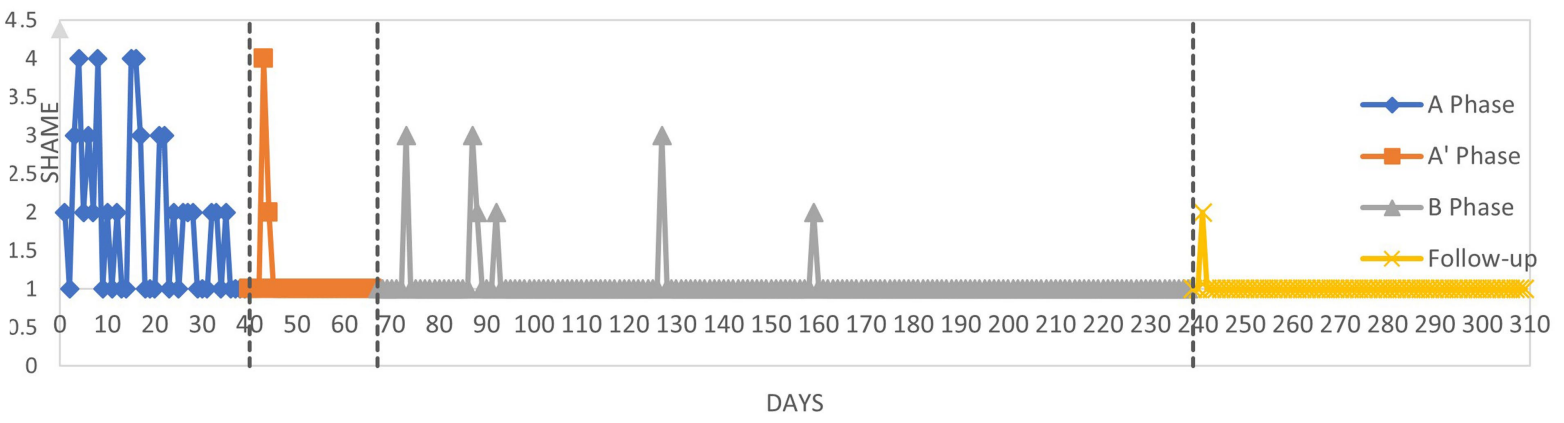
Daily reports of shame across phases: “How often I have felt ashamed today?”.

Figures 2–4 graphically display the participant’s weekly measures of self-stigma and self-compassion and daily reports of shame.

#### 3.2.1 Visual inspection

Visually, self-compassion increased while shame decreased across the pure baseline (A) and the conceptualization phase (A’). Regarding self-compassion during the active treatment period (B), there was some variability around scores of 1.5 to 1.8 until week 21 followed by a consistent increase of scores for 5 weeks up to 3 which then decreased slightly to reach a score of 2.2 at the end of the active treatment phase. Self-compassion increased again during the follow-up period and ranged between 2.5 and 2.8 during the 5-week follow-up period suggesting that the amelioration of self-compassion during the intervention was maintained post-therapy.

Regarding self-stigma scores, the trend is less straightforward than for self-compassion. Indeed, there is a large variability of self-stigma scores during the pure baseline period (A) making the changes in self-stigma across the subsequent phases difficult to interpret. In the conceptualization phase (A’), self-stigma progressively decreased but the variability of scores persisted, suggesting that the conceptualization phase had a small effect on self-stigma. During the active therapeutic phase (B) and the follow-up period, self-stigma scores continued to show great variations.

Daily reports of shame during the pure baseline period (A) indicate that Julian frequently felt ashamed. At the beginning of the conceptualization (A’), Julian reported a high level of shame which showed a sudden subsequent decrease. During the start of the active treatment phase (B), shame was reported to be more present than for the rest of this phase suggesting a decrease of shame during the CFT phase as compared to the baseline. This reduction of shame was maintained during the follow-up period.

#### 3.2.2 Statistical analyses

Tau-U was calculated to investigate changes across the different phases. Results are given in Table 6 and interpreted according to Vannest and Ninci (78): A 0.20 improvement can be considered a small change, 0.20 to 0.60 a moderate change, 0.60 to 0.80 a large change, and above 0.80 a large to very large change. Statistical analysis indicates a large increase in self-compassion between the baseline (including the conceptualization phase) and the active treatment phase (Tau-U = 0.74). If the baseline phase is considered without the conceptualization phase, the change is very large (Tau-U = 0.99). These results suggest that self-compassion increases during the conceptualization phase even though there is no active compassionate mind training during the conceptualization sessions. There is no difference between the intervention phase and the follow-up (Tau-U = 0.00) suggesting that the amelioration of self-compassion is maintained after the intervention. Regarding self-stigma, there is a moderate decrease of self-stigma between the baseline (including the conceptualization phase) and the active treatment phase (Tau-U = 0.32). In contrast, when we compare the baseline without the conceptualization phase with the intervention phase (which includes the conceptualization), there is no change in self-stigma (Tau-U = −0.09). There is no change in self-stigma between the intervention and follow-up (Tau-U = −0.19). Regarding daily shame, there is moderate decrease in shame between the baseline including the conceptualization phase and the active treatment phase (Tau-U = 0.31) and a moderate decrease between the baseline (without the conceptualization phase) and the intervention phase including the conceptualization (Tau-U = 0.51).

**Table 6:** Tau calculations for the weekly measures of self-compassion and self-stigma.

	AA’ versus B	A versus A’B	B versus Follow-up^**^	A’B versus Follow-up^**^
Weekly self-compassion^*^	0.74	0.99	0.00	−0.08
Weekly self-stigma^*^	0.32	−0.09	−0.08	−0.19
Daily shame	0.31	0.51	0.02	0.03

^*^Tau-U was used in order to include baseline trend correction. ^**^If intervention effects are maintained over time, a Tau/TAU-U close to zero is expected.

The percentages of goals obtained were calculated for both self-compassion and shame (Table 7). Between baseline (including the conceptualization phase) and the active intervention phase, 64.91% of goal in self-compassion was obtained. When we include the conceptualization phase as part of the intervention, the percentage of goal is almost the same (62.49%). Across the intervention phase and the follow-up phase the percentage of goal obtained is very high: 143.10%. Considering that the goal was set as the benchmark between low self-compassion to moderate self-compassion, this result suggests that during the follow-up, self-compassion was consistently higher that the “moderate” cut-off. Regarding daily shame reports, the percentage of goal obtained is also very similar whether we include the conceptualization in the baseline or the intervention phase, respectively 90.21 and 92.51%. In the follow-up, the percentage of goal remained high suggesting that the effects of the intervention on daily shame reports remained stable during the follow-up.

**Table 7:** Percentage of goal obtained for weekly self-compassion and daily shame.

	AA’ versus B	A versus A’B	B versus Follow-up	A’B versus Follow-up
Self-compassion	Effect size estimate: 64.91 Standard error: 10.91 95% CI: [43.53, 86.29]	Effect size estimate: 62.49 Standard error: 8.61 95% CI: [45.61, 79.37]	Effect size estimate: 143.10 Standard error: 43.37 95% CI: [58.10, 228.11]	Effect size estimate: 136.02 Standard error: 33.07 95% CI: [71.20, 200.85]
Shame	Effect size estimate: 90.91 Standard error: 27.12 95% CI: [37.75, 144.07]	Effect size estimate: 92.51 Standard error: 25.30 95% CI: [42.92, 142.09]	Effect size estimate: 76.69 Standard error: 58.38 95% CI: [−37.74, 191.12]	Effect size estimate: 81.43 Standard error: 52.28 95% CI: [−21.04, 183.89]

## 4 Discussion

In this study, as recommended by recent literature referring to healthcare and rehabilitation, we used a single case pre-experimental design to evaluate the feasibility and efficacy of CFT for reducing self-stigma in an autistic adult (47). To do so, we investigated whether and how scores of self-compassion, self-stigma and shame measures evolved through a baseline period, a conceptualization phase, CFT and a follow-up period.

First of all, we found that CFT was highly acceptable and feasible with this autistic client. Indeed, Julian was very diligent with session attendance and in-between sessions practices, with more than a hundred entries in his log of home practices across the intervention and follow-up phases. This result is important given that barriers to accessing care are numerous in this population, including clinician attitudes about autism and the importance of flexibility for the individualization of treatment (79). In addition, the individual format of the intervention allowed the therapist to adapt the sessions to better fit the needs of the participant. For example, the client’s interests and passions were used in the therapeutic tasks, which may have contributed to increase the acceptability of CFT (60).

In terms of the efficacy measures, self-compassion increased throughout the conceptualization, CFT and follow-up phases. More specifically, self-compassion increased while shame decreased across the baseline and the conceptualization phase. This is consistent with the de-shaming function of case conceptualization in CFT (80). Indeed, the collaborative creation of a common understanding of the client’s difficulties through a compassionate lens may have been effective in reducing shame (i.e., “we did not choose to have a brain that functions this way and can create difficult emotions and ruminations, nor did we choose the context in which we grew up”). In other words, the idea that “it is not my fault” brought about in the conceptualization phase might explain the reduction of shame found during this phase as opposed to the pure baseline phase.

In contrast to self-compassion, the self-stigma measure showed high variability during the baseline, making subsequent results and changes difficult to interpret. A moderate decrease in self-stigma was nevertheless found between the baseline and the intervention phase, which was maintained at follow-up. Similar results were found on the daily reports of shame, consistent with the idea that shame is strongly associated with self-stigma in autistic and non-autistic people) (27, 81). The moderate changes in self-stigma might be explained by different factors. First, internalized stigma is the result of frequent and repeated exposure to stigmatizing attitudes and experiences of discrimination. Thus, although self-compassion significantly increased during treatment and this change was maintained at follow up, self-stigma may take more time to change, especially because public stigma remains prevalent. Relatedly, it is worth noting that, as the therapy advanced and self-compassion increased, the participant progressively exposed himself to social situations that he avoided before, momentarily increasing his anxiety, and feelings of inadequacy. Indeed, increased social exposure to a non-autistic world comes with a high risk to face negative attitudes from others (64), and this might explain the results found in our study. Another explanation is related to the scale that was used to assess self-stigma, i.e., the ISMI (54). Indeed, the ISMI was validated for measuring self-stigma related to psychiatric disorders, thus it is possible that the ISMI did not fully capture autism-related self-stigma.

Overall, our results suggest that self-compassion increased following CFT, but self-stigma decreased only moderately. We speculate that this is due to the persistence of autism-related stigma in our society. Hence, in addition to tackling self-stigma, it is crucial to address and fight autism-related public stigma within our societies. To achieve this, it is pivotal to implement anti-stigma programs targeting the general public, families of autistic individuals, teachers, and health practitioners. However, existing programs are scare, have shown limited effectiveness so far (82), and changing public attitudes takes effort and time. Some studies focusing on anti-stigma programs for autism have shown promising results but have been constrained by methodological limitations, such as short-term effects and lack of behavioral measures (83–86). Considering these challenges, in addition to working on reducing public stigma, this study demonstrates how CFT may decrease autism-related self-stigma and thus addresses one of the facets of the stigmatization process. By doing so, CFT could contribute to reduce the negative health consequences associated with stigma in autistic individuals.

From a qualitative standpoint, Julian reported that the therapy helped him become less self-critical and self-stigmatizing, suggesting that, from a subjective perspective, self-stigma decreased. Indeed, during the last sessions of the therapy, Julian shared that writing his novel meant that he was embracing who he was, i.e., “*I accept who I am*.” He shared that his novel was now part of his life and contributed to “*his acceptance of his difference*,” that is, he accepted and embraced his autistic identity instead of camouflaging it in most occasions or avoiding social situations. Consistently, he mentioned “*I show myself as I am, I have no more desire to be a chameleon because I know it’s destructive, I have no more need to hide*” “*I’m naked, camouflage falls off, I am now more sensitive to my environment, I have not fallen back into the idea of being perfect, I have my flaws, they make up my personality*.” He also noticed that he was more aware of his emotions “*My awareness and acuity when it’s happening improved, there is now a real synchronization when it’s happening, so I can see what I can use right away to be helpful*.” Furthermore, Julian reported “*I can notice I’ve been hurt by someone and I’m able to ask for an apology, compassion goes in both directions*,” suggesting that his self-compassion, but also compassion for others increased following CFT. Hence, the self-reported decrease in self-stigma through self-compassion seems to have allowed him to hide and avoid less while decreasing his use of camouflaging behaviors. In addition, he acknowledged that CFT also helped him become more aware of his emotions, a finding in line with previous studies using CFT (87). Both results are clinically important given that camouflaging, social isolation, and emotion dysregulation have been found to be involved in the high rates depression, anxiety and suicidality in autistic adults (7, 65, 88, 89).

As a pilot clinical study, this research presents with limitations. Firstly, the shift between the baseline and the intervention phase is not clear-cut, given that the conceptualization phase can be either seen as part of the baseline or the intervention. This is particularly the case in an A-B single case design such as ours. Further research should aim at replicating our results and explore changes across the conceptualization and active treatment phases possibly with a larger sample of autistic individuals. Moreover, to decrease the load on the participant, only selected measures of self-stigma, self-compassion and shame were used. However, given the link between self-stigma and mental-health, future studies should consider using measures of psychopathology (e.g., depression and anxiety scales) and quality of life to explore the effects of the intervention more broadly. Furthermore, because autism can take many shapes, further research should include larger scale studies involving a wide variety of autistic clients (e.g., multiple baseline SCED design and randomized controlled studies). Finally, research should investigate the types of adaptations of CFT required for autistic clients more broadly.

In conclusion, the present study adds to the CFT literature by demonstrating its acceptability and preliminary efficacy in reducing self-stigma in autistic adults. Given that self-stigma seems to be involved in the diminished social functioning of autistic adults as well as in the maintenance of co-occurring disorders, our results are particularly promising and point to the need to conduct more large-scale studies on CFT in autistic adults.

## Data availability statement

The raw data supporting the conclusions of this article will be made available by the authors, without undue reservation.

## Ethics statement

Ethical approval was assigned by the French Ethics Committee (Comité de Protection des Personnes Nord Ouest II) - (2022-A02501-42). The studies were conducted in accordance with the local legislation and institutional requirements. The participants provided their written informed consent to participate in this study.

## Author contributions

MR: Conceptualization, Data curation, Formal analysis, Funding acquisition, Investigation, Methodology, Resources, Writing – original draft, Software, Visualization. AK-P: Methodology, Writing – review & editing, Supervision. RM: Methodology, Writing – review & editing. OR: Writing – review & editing. LW: Conceptualization, Supervision, Writing – review & editing, Project administration.

## References

[1] Volkmar FR Reichow B. Autism in DSM-5: progress and challenges. Mol Autism. (2013) 4:13. doi: 10.1186/2040-2392-4-13, PMID: 23675688 PMC3716827

[2] Zeidan J Fombonne E Scorah J Ibrahim A Durkin MS Saxena S. Global prevalence of autism: a systematic review update. Autism Res. (2022) 15:778–90. doi: 10.1002/aur.2696, PMID: 35238171 PMC9310578

[3] Hollocks MJ Lerh JW Magiati I Meiser-Stedman R Brugha TS. Anxiety and depression in adults with autism spectrum disorder: a systematic review and meta-analysis. Psychol Med. (2019) 49:559–72. doi: 10.1017/S0033291718002283 30178724

[4] Bejerot S Mörtberg E. Do autistic traits play a role in the bullying of obsessive-compulsive disorder and social phobia sufferers? Psychopathology. (2009) 42:170–6. doi: 10.1159/000207459, PMID: 19276643

[5] Hebron J Humphrey N Oldfield J. Vulnerability to bullying of children with autism spectrum conditions in mainstream education: a multi-informant qualitative exploration. J Res Spec Educ Needs. (2015) 15:185–93. doi: 10.1111/1471-3802.12108

[6] Zablotsky B Black LI Maenner MJ Schieve LA Blumberg SJ. Estimated prevalence of autism and other developmental disabilities following questionnaire changes in the 2014 national health interview survey. Natl Health Stat Rep. (2015) 2015:1–20. 26632847

[7] Cage E Di Monaco J Newell V. Experiences of autism acceptance and mental health in autistic adults. J Autism Dev Disord. (2018) 48:473–84. doi: 10.1007/s10803-017-3342-7, PMID: 29071566 PMC5807490

[8] Jacoby EC Walton K Guada J. Community perspectives on adults with autism Spectrum disorder. Occup Ther Ment Health. (2019) 35:72–91. doi: 10.1080/0164212X.2018.1507774

[9] Perry E Mandy W Hull L Cage E. Understanding camouflaging as a response to autism-related stigma: a social identity theory approach. J Autism Dev Disord. (2022) 52:800–10. doi: 10.1007/s10803-021-04987-w, PMID: 33788076 PMC8813820

[10] Cage E Troxell-Whitman Z. Understanding the reasons, contexts and costs of camouflaging for autistic adults. J Autism Dev Disord. (2019) 49:1899–911. doi: 10.1007/S10803-018-03878-X, PMID: 30627892 PMC6483965

[11] Mazumder R Thompson-Hodgetts S. Stigmatization of children and adolescents with autism spectrum disorders and their families: a scoping study. Rev J Autism Dev Disord. (2019) 6:96–107. doi: 10.1007/s40489-018-00156-5

[12] Pescosolido BA. The public stigma of mental illness: what do we think; what do we know; what can we prove? J Health Soc Behav. (2013) 54:1–21. doi: 10.1177/0022146512471197 23325423 PMC4437625

[13] Huang CH Li SM Shu BC. Exploring the relationship between illness perceptions and negative emotions in relatives of people with schizophrenia within the context of an affiliate stigma model. J Nurs Res. (2016) 24:217–23. doi: 10.1097/jnr.0000000000000124, PMID: 26588452

[14] Corrigan PW Watson AC. Understanding the impact of stigma on people with mental illness. World Psychiatry. (2002) 1:16. 16946807 PMC1489832

[15] Gilbert P Miles JNV. Sensitivity to social put-down: It’s relationship to perceptions of social rank, shame, social anxiety, depression, anger and self-other blame. Personal Individ Differ. (2000) 29:757–74. doi: 10.1016/S0191-8869(99)00230-5

[16] Gilbert P. Introducing compassion-focused therapy. Adv Psychiatr Treat. (2009) 15:199–208. doi: 10.1192/apt.bp.107.005264

[17] Gilbert P. The relationship of shame, social anxiety and depression: the role of the evaluation of social rank. Clin Psychol Psychother. (2000) 7:174–89. doi: 10.1002/1099-0879(200007)7:3<174::AID-CPP236>3.0.CO;2-U

[18] Gilbert P. The evolution of social attractiveness and its role in shame, humiliation, guilt and therapy. Br J Med Psychol. (1997) 70:113–47. doi: 10.1111/j.2044-8341.1997.tb01893.x 9210990

[19] Gilbert P. What is shame? Some core issues and controversies In: Gilbert P Andrews B, editors. Shame: Interpersonal Behavior, Psychopathology, and Culture. Oxford University Press. (1998) 3–38.

[20] Tangney JP Wagner P Gramzow R. Proneness to shame, proneness to guilt, and psychopathology. J Abnorm Psychol. (1992) 101:469–78. doi: 10.1037/0021-843X.101.3.469 1500604

[21] Schibalski JV Müller M Ajdacic-Gross V Vetter S Rodgers S Oexle N. Stigma-related stress, shame and avoidant coping reactions among members of the general population with elevated symptom levels. Compr Psychiatry. (2017) 74:224–30. doi: 10.1016/j.comppsych.2017.02.001, PMID: 28236772

[22] Bachmann CJ Höfer J Kamp-Becker I Küpper C Poustka L Roepke S. Internalised stigma in adults with autism: a German multi-center survey. Psychiatry Res. (2019) 276:94–9. doi: 10.1016/j.psychres.2019.04.023 31030006

[23] Botha M Dibb B Frost DM. “Autism is me”: an investigation of how autistic individuals make sense of autism and stigma. Disabil Soc. (2022) 37:427–53. doi: 10.1080/09687599.2020.1822782

[24] Dubreucq J Plasse J Gabayet F Faraldo M Blanc O Chereau I. Self-stigma in serious mental illness and autism spectrum disorder: results from the REHABase national psychiatric rehabilitation cohort. Eur Psychiatry. (2020) 63:e13. doi: 10.1192/j.eurpsy.2019.12, PMID: 32093806 PMC7315867

[25] McDonald TAM. Correction to: discriminative and criterion validity of the autism Spectrum identity scale (ASIS). J Autism Dev Disord. (2020) 50:340–1. doi: 10.1007/s10803-019-04228-1 31571068

[26] Shtayermman O. An exploratory study of the stigma associated with a diagnosis of Asperger’s syndrome: the mental health impact on the adolescents and young adults diagnosed with a disability with a social nature. J Hum Behav Soc Environ. (2009) 19:298–313. doi: 10.1080/10911350902790720

[27] Riebel M Bureau R Rohmer O Clément C Weiner L. Self-compassion as an antidote to self-stigma and shame in autistic adults. (Submitted). 10.1177/13623613251316965 39959965

[28] Wong CCY Knee CR Neighbors C Zvolensky MJ. Hacking stigma by loving yourself: a mediated-moderation model of self-compassion and stigma. Mindfulness. (2019) 10:415–33. doi: 10.1007/s12671-018-0984-2

[29] Neff KD Kirkpatrick KL Rude SS. Self-compassion and adaptive psychological functioning. J Res Pers. (2007) 41:139–54. doi: 10.1016/J.JRP.2006.03.004

[30] Brown L Houston EE Amonoo HL Bryant C. Is self-compassion associated with sleep quality? A meta-analysis. Mindfulness. (2021) 12:82–91. doi: 10.1007/s12671-020-01498-0

[31] Cleare S Gumley A O’Connor RC. Self-compassion, self-forgiveness, suicidal ideation, and self-harm: a systematic review. Clin. Psychol. Psychother. (2019) 26:511–30. doi: 10.1002/cpp.2372, PMID: 31046164

[32] MacBeth A Gumley A. Exploring compassion: a meta-analysis of the association between self-compassion and psychopathology. Clin Psychol Rev. (2012) 32:545–52. doi: 10.1016/j.cpr.2012.06.003 22796446

[33] Zessin U Dickhäuser O Garbade S. The relationship between self-compassion and well-being: a meta-analysis. Appl Psychol Health Well Being. (2015) 7:340–64. doi: 10.1111/aphw.12051 26311196

[34] Hilbert A Braehler E Schmidt R Lowe B Hauser W Zenger M. Self-compassion as a resource in the self-stigma process of overweight and obese individuals. Obes Facts. (2015) 8:293–301. doi: 10.1159/000438681, PMID: 26422226 PMC5644803

[35] Skinta MD Fekete EM Williams SL. HIV-stigma, self-compassion, and psychological well-being among gay men living with HIV. Stigma Health. (2019) 4:179–87. doi: 10.1037/sah0000133

[36] Heath PJ Brenner RE Lannin DG Vogel DL. Self-compassion moderates the relationship of perceived public and anticipated self-stigma of seeking help. Stigma Health. (2018) 3:65. doi: 10.1037/sah0000072

[37] Cai RY Gibbs V Love A Robinson A Fung L Brown L. “Self-compassion changed my life”: the self-compassion experiences of autistic and non-autistic adults and its relationship with mental health and psychological wellbeing. J Autism Dev Disord. (2022) 53:1066–81. doi: 10.1007/s10803-022-05668-y, PMID: 35904649

[38] Galvin J Richards G. The indirect effect of self-compassion in the association between autistic traits and anxiety/depression: a cross-sectional study in autistic and non-autistic adults. Autism. (2022) 27:1256–70. doi: 10.1177/13623613221132109 36341962

[39] Cai RY Brown L. Cultivating self-compassion to improve mental health in autistic adults. Autism Adulthood. (2021) 3:230–7. doi: 10.1089/aut.2020.0034, PMID: 36605368 PMC8992903

[40] Cai RY Love A Robinson A Gibbs V. The inter-relationship of emotion regulation, self-compassion, and mental health in autistic adults. Autism in Adulthood. (2023) 5:335–42. doi: 10.1089/aut.2022.0068 PMC10468559 37663445

[41] Goffnett J Liechty JM Kidder E. Interventions to reduce shame: a systematic review. J Behav Cogn Ther. (2020) 30:141–60. doi: 10.1016/J.JBCT.2020.03.001

[42] Gilbert P. Social mentalities: a biopsychosocial and evolutionary approach to social relationships In: Baldwin MW, editor. Interpersonal Cognition. New York: The Guilford Press (2005). 299–333.

[43] Craig C Hiskey S Spector A. Compassion focused therapy: a systematic review of its effectiveness and acceptability in clinical populations. Expert Rev Neurother. (2020) 20:385–400. doi: 10.1080/14737175.2020.1746184, PMID: 32196399

[44] Kirby JN Gilbert P. Commentary regarding Wilson et al. (2018) “effectiveness of ‘self-compassion’ related therapies: a systematic review and Meta-analysis.” all is not as it seems. Mindfulness. (2019) 10:1006–16. doi: 10.1007/s12671-018-1088-8

[45] Riebel M Rohmer O Weiner L. Compassion focused therapy (CFT) for the reduction of the self-stigma of mental disorders: The COMpassion for psychiatric disorders and self-stigma (COMPASS) study protocol for a randomized controlled study. Trials. (2023) 24:393. doi: 10.1186/s13063-023-07393-y 37309006 PMC10258933

[46] Mason D Acland J Stark E Happé F Spain D. Compassion-focused therapy with autistic adults. Front Psychol. (2023) 14:1267968. doi: 10.3389/fpsyg.2023.1267968, PMID: 37965655 PMC10641016

[47] Krasny-Pacini A Evans J. Single-case experimental designs to assess intervention effectiveness in rehabilitation: a practical guide. Ann Phys Rehabil Med. (2018) 61:164–79. doi: 10.1016/j.rehab.2017.12.002 29253607

[48] Ritsher JB Phelan JC. Internalized stigma predicts erosion of morale among psychiatric outpatients. Psychiatry Res. (2004) 129:257–65. doi: 10.1016/j.psychres.2004.08.003, PMID: 15661319

[49] Linehan MM. Skills Training Manual for Treating Borderline Personality Disorder. New York: Guilford Press (1993).

[50] Gast DL Ledford JR. Single Case Research Methodology. 3rd ed Routledge (2018).

[51] Neff KD. The development and validation of a scale to measure self-compassion. Self Identity. (2023) 2:223–50. doi: 10.1080/15298860309027

[52] Raes F Pommier E Neff KD Van Gucht D. Construction and factorial validation of a short form of the self-compassion scale. Clin Psychol Psychother. (2011) 18:250–5. doi: 10.1002/cpp.702 21584907

[53] Kotsou I Leys C. Self-compassion scale (SCS): psychometric properties of the French translation and its relations with psychological well-being, affect and depression. PLoS One. (2016) 11:e0152880. doi: 10.1371/JOURNAL.PONE.0152880, PMID: 27078886 PMC4831759

[54] Kratochwill TR Hitchcock JH Horner RH Levin JR Odom SL Rindskopf DM. Single-Case Intervention Research Design Standards. Remedial Spec. Educ. (2013) 34:26–38. doi: 10.1177/0741932512452794

[55] Kratochwill T. R. Hitchcock J. Horner R. H. Levin J. R. Odom S. L. Rindskopf D. M.. (2010). Single-Case Designs Technical Documentation. What Works Clearinghouse. Available at: http://ies.ed.gov/ncee/wwc/pdf/wwc_scd.pdf.

[56] Parker RI Vannest KJ Davis JL Sauber SB. Combining nonoverlap and trend for single-case research: tau-U. Behav Ther. (2011) 42:284–99. doi: 10.1016/J.BETH.2010.08.006, PMID: 21496513

[57] Pustejovsky JE. Procedural sensitivities of effect sizes for single-case designs with directly observed behavioral outcome measures. Psychol Methods. (2019) 24:217–35. doi: 10.1037/met0000179 29911874

[58] Ferron J Goldstein H Olszewski A Rohrer L. Indexing effects in single-case experimental designs by estimating the percent of goal obtained. Evid Based Commun Assess Interv. (2020) 14:6–27. doi: 10.1080/17489539.2020.1732024

[59] Bemmouna D Coutelle R Weibel S Weiner L. Feasibility, acceptability and preliminary efficacy of dialectical behavior therapy for autistic adults without intellectual disability: a mixed methods study. J Autism and Dev Disord. (2021) 52:4337–54. doi: 10.1007/S10803-021-05317-W, PMID: 34626285 PMC8501315

[60] Keenan EG Gurba AN Mahaffey B Kappenberg CF Lerner MD. Leveling up dialectical behavior therapy for autistic individuals with emotion dysregulation: clinical and personal insights. Autism Adulthood. (2023). doi: 10.1089/AUT.2022.0011 PMC10902278 38435330

[61] Gilbert P. A brief outline of the evolutionary approach for compassion focused therapy. EC Psychol Psychiatry. (2017) 3:218–27.

[62] Gilbert P. Compassion: from its evolution to a psychotherapy. Front Psychol. (2020) 11:586161. doi: 10.3389/fpsyg.2020.586161, PMID: 33362650 PMC7762265

[63] Hull L Petrides KV Allison C Smith P Baron-Cohen S Lai MC. “Putting on my best Normal”: social camouflaging in adults with autism Spectrum conditions. J Autism Dev Disord. (2017) 47:2519–34. doi: 10.1007/S10803-017-3166-5, PMID: 28527095 PMC5509825

[64] Bureau R Riebel M Weiner L Coutelle R Dachez J Clément C. French Validation of the Camouflaging Autistic Traits Questionnaire (CAT-Q)s. J Autism Dev Disord. (2023) 1–10. 10.1007/s10803-023-06048-w 37349595

[65] Cook J Hull L Crane L Mandy W. Camouflaging in autism: a systematic review. Clin Psychol Rev. (2021) 89:102080. doi: 10.1016/J.CPR.2021.102080, PMID: 34563942

[66] Dudley R Kuyken W Padesky CA. Disorder specific and trans-diagnostic case conceptualisation. Clin Psychol Rev. (2011) 31:213–24. doi: 10.1016/J.CPR.2010.07.005, PMID: 20813444

[67] Irons C Heriot-Maitland C. Compassionate mind training: an 8-week group for the general public. Psychol Psychother Theory Res Pract. (2021) 94:443–63. doi: 10.1111/PAPT.12320 33222375

[68] Gilbert P. An introduction to compassion focused therapy in cognitive behavior therapy. Int. J. Cogn. Ther. (2010) 3:97–112.

[69] Laborde S Allen MS Borges U Dosseville F Hosang TJ Iskra M. Effects of voluntary slow breathing on heart rate and heart rate variability: a systematic review and a meta-analysis. Neurosci Biobehav Rev. (2022) 138:104711. doi: 10.1016/J.NEUBIOREV.2022.104711, PMID: 35623448

[70] Di Bello M Ottaviani C Petrocchi N. Compassion is not a benzo: distinctive associations of heart rate variability with its empathic and action components. Front Neurosci. (2021) 15:617443. doi: 10.3389/FNINS.2021.617443/BIBTEX 33776635 PMC7994334

[71] Petrocchi N Cheli S. The social brain and heart rate variability: implications for psychotherapy. Psychol Psychother Theory Res Pract. (2019) 92:208–23. doi: 10.1111/PAPT.12224, PMID: 30891894

[72] Gilbert P. Mindful compassion. Hachette UK (2013).

[73] Riebel M Weiner L. Feasibility and acceptability of group compassion-focused therapy to treat the consequences of childhood maltreatment in people with psychiatric disorders in France. J Nerv Ment Dis. (2023) 211:393–401. doi: 10.1097/NMD.0000000000001603, PMID: 37040141

[74] Bell T Montague J Elander J Gilbert P. “A definite feel-it moment”: embodiment, externalisation and emotion during chair-work in compassion-focused therapy. Couns Psychother Res. (2020) 20:143–53. doi: 10.1002/capr.12248

[75] Lucre K Clapton N. The compassionate kitbag: a creative and integrative approach to compassion-focused therapy. Psychol Psychother. (2021) 94:497–516. doi: 10.1111/papt.12291, PMID: 32639097

[76] Hayes SC Strosahl KD Wilson KG. Acceptance and commitment therapy In: Arthur F, editor. Encyclopedia Cognitive Behavior Therapy, vol. 6. New York: Guilford Press (1999)

[77] Steindl SR Kirby JN Tellegan C. Motivational interviewing in compassion-based interventions: theory and practical applications. Clin Psychol. (2018) 22:265–79. doi: 10.1111/cp.12146

[78] Vannest KJ Ninci J. Evaluating intervention effects in single-case research designs. J Couns Dev. (2015) 93:403–11. doi: 10.1002/JCAD.12038

[79] Brede J Cage E Trott J Palmer L Smith A Serpell L. “We have to try to find a way, a clinical bridge” - autistic adults’ experience of accessing and receiving support for mental health difficulties: a systematic review and thematic meta-synthesis. Clin Psychol Rev. (2022) 93:102131. doi: 10.1016/j.cpr.2022.102131, PMID: 35180632

[80] Heriot-Maitland C Levey V. A case report of compassion-focused therapy for distressing voice-hearing experiences. J Clin Psychol. (2021) 77:1821–35. doi: 10.1002/jclp.23211, PMID: 34252979

[81] Hasson-Ohayon I Ehrlich-Ben Or S Vahab K Amiaz R Weiser M Roe D. Insight into mental illness and self-stigma: the mediating role of shame proneness. Psychiatry Res. (2012) 200:802–6. doi: 10.1016/J.PSYCHRES.2012.07.038, PMID: 22889545

[82] Godeau E Vignes C Sentenac M Ehlinger V Navarro F Grandjean H. Improving attitudes towards children with disabilities in a school context: a cluster randomized intervention study. Dev Med Child Neurol. (2010) 52:e236–42. doi: 10.1111/J.1469-8749.2010.03731.X, PMID: 20646032

[83] Campbell JM Ferguson JE Herzinger CV Jackson JN Marino CA. Combined descriptive and explanatory information improves peers’ perceptions of autism. Res Dev Disabil. (2004) 25:321–39. doi: 10.1016/j.ridd.2004.01.005, PMID: 15193668

[84] Ranson NJ Byrne MK. Promoting peer acceptance of females with higher-functioning autism in a mainstream education setting: a replication and extension of the effects of an autism anti-stigma program. J Autism Dev Disord. (2014) 44:2778–96. doi: 10.1007/s10803-014-2139-1 24816870

[85] Schachter HM Girardi A Ly M Lacroix D Lumb AB Van Berkom J. Effects of school-based interventions on mental health stigmatization: a systematic review. Child Adolesc Psychiatry Ment Health. (2008) 2:18. doi: 10.1186/1753-2000-2-18, PMID: 18644150 PMC2515285

[86] Staniland JJ Byrne MK. The effects of a multi-component higher-functioning autism anti-stigma program on adolescent boys. J Autism Dev Disord. (2013) 43:2816–29. doi: 10.1007/s10803-013-1829-4 23619951

[87] Gilbert P Simos G. (Eds.). Compassion focused therapy: Clinical practice and applications. Routledge (2022).

[88] Cassidy S Bradley L Shaw R Baron-Cohen S. Risk markers for suicidality in autistic adults. Mol Autism. (2018) 9:42. doi: 10.1186/s13229-018-0226-4, PMID: 30083306 PMC6069847

[89] Conner CM Golt J Righi G Shaffer R Siegel M Mazefsky CA. A comparative study of suicidality and its association with emotion regulation impairment in large ASD and US census-matched samples. J Autism Dev Disord. (2020) 50:3545–60. doi: 10.1007/s10803-020-04370-1, PMID: 31939083 PMC7893480

